# Cost-effectiveness analysis of bortezomib in combination with rituximab, cyclophosphamide, doxorubicin, vincristine and prednisone (VR-CAP) in patients with previously untreated mantle cell lymphoma

**DOI:** 10.1186/s12885-016-2633-2

**Published:** 2016-08-04

**Authors:** Marjolijn van Keep, Kerry Gairy, Divyagiri Seshagiri, Pushpike Thilakarathne, Dawn Lee

**Affiliations:** 1 BresMed, Arthur van Schendelstraat 650, 3511MJ Utrecht, The Netherlands; 2 Janssen-Cilag, 50-100 Holmers Farm Way, High Wycombe, HP12 4EG UK; 3 Janssen-Cilag, Johnson & Johnson Platz 1, 41470 Neuss, Germany; 4 Janssen-Cilag, Turnhoutseweg 30, B-2340 Beerse, Belgium; 5 BresMed, 84 Queen Street, Sheffield, S1 2DW UK

**Keywords:** Bortezomib, Mantle cell lymphoma, Cost effectiveness, VR-CAP, R-CHOP

## Abstract

**Background:**

Mantle cell lymphoma (MCL) is a rare and aggressive form of non-Hodgkin’s lymphoma. Bortezomib is the first product to be approved for the treatment of patients with previously untreated MCL, for whom haematopoietic stem cell transplantation is unsuitable, and is used in combination with rituximab, cyclophosphamide, doxorubicin, vincristine and prednisone (VR-CAP). The National Institute of Health and Care Excellence recently recommended the use of VR-CAP in the UK following a technology appraisal. We present the cost effectiveness analysis performed as part of that assessment: VR-CAP versus the current standard of care regimen of rituximab, cyclophosphamide, doxorubicin, vincristine and prednisone (R-CHOP) in a UK setting.

**Methods:**

A lifetime economic model was developed with health states based upon line of treatment and progression status. Baseline patient characteristics, dosing, safety and efficacy were based on the LYM-3002 trial. As overall survival data were immature, survival was modelled by progression status, and post-progression survival was assumed equal across arms. Utilities were derived from LYM-3002 and literature, and standard UK cost sources were used.

**Results:**

Treatment with VR-CAP compared to R-CHOP gave an incremental quality-adjusted life year (QALY) gain of 0.81 at an additional cost of £16,212, resulting in a base case incremental cost-effectiveness ratio of £20,043. Deterministic and probabilistic sensitivity analyses showed that treatment with VR-CAP was cost effective at conventional willingness-to-pay thresholds (£20,000–£30,000 per QALY).

**Conclusions:**

VR-CAP is a cost-effective option for previously untreated patients with MCL in the UK.

**Electronic supplementary material:**

The online version of this article (doi:10.1186/s12885-016-2633-2) contains supplementary material, which is available to authorized users.

## Background

Mantle cell lymphoma (MCL) is a rare, incurable and aggressive sub-type of non-Hodgkin’s lymphoma (NHL), accounting for approximately 6 % of all NHL cases [1]. The incidence of MCL in the UK is 0.9 per 100,000 [1]. The general pattern of disease progression in MCL is one of relapse and remission, with each relapse becoming more difficult to treat, and the depth and durability of any subsequent remissions achieved invariably inferior to those achieved with first-line treatment [2–6].

In patients first presenting with aggressive disease requiring treatment, the initial treatment decision is whether patients are suitable for high-intensity induction therapy, to be followed by haematopoietic stem cell transplantation (HSCT). There are no strict criteria against which patients are assessed; rather, haematologists will assess eligibility on a patient-by-patient basis, taking into account factors such as patient age, performance status and disease prognosis, disease severity, co-morbidities, and clinical risk [2, 5–10].

For patients who are not eligible for high-intensity induction therapy, that is those for whom HSCT is unsuitable, there had been no licensed induction therapy regimens prior to bortezomib. Rituximab, cyclophosphamide, doxorubicin, vincristine and prednisone (R-CHOP) became the preferred first-line induction therapy in UK clinics because the large scale European MCL Elderly trial [11] demonstrated a survival benefit for R-CHOP when compared with rituximab in combination with fludarabine and cyclophosphamide (R-FC). Alternative rituximab-based chemotherapy induction regimens are also administered in the first-line setting, but usually only for the frailest of patients considered unsuitable for R-CHOP therapy; while alternatives are considered to be associated with lower toxicity, the evidence base supporting their use is considerably weaker [12]. Median progression-free survival (PFS) associated with chemotherapy is less than 2 years, and median overall survival (OS) is less than 5 years [10, 13–19].

Bortezomib is the first product to be licensed for the treatment of patients with previously untreated MCL for whom HSCT is unsuitable. Bortezomib is administered in combination with the rituximab, cyclophosphamide, doxorubicin, prednisone backbone familiar to clinicians as part of the R-CHOP regimen. A randomised, open-label, multicentre Phase III study (LYM-3002) comparing bortezomib, rituximab, cyclophosphamide, doxorubicin and prednisolone (VR-CAP) to R-CHOP showed a significant improvement in PFS (24.7 versus 14.4 months; hazard ratio [HR] = 0.63, *p* < 0.001) based on the primary assessment of PFS by the independent review committee (IRC) [20]. Duration of overall response for VR-CAP was more than double that of R-CHOP (median of 36.5 versus 15.1 months), resulting in an increase in the treatment free interval (TFI) of almost 20 months versus R-CHOP (median of 40.6 versus 20.5 months; HR = 0.50, *p* < 0.001) [20].

There have been no previous technology appraisals by the National Institute of Health and Care Excellence (NICE) within MCL; other therapies that are frequently used such as bendamustine and temsirolimus did not go through the UK health technology assessment (HTA) process due to lack of marketing authorisation approval and manufacturer non-submission, respectively. To gain NICE recommendation for VR-CAP, the cost effectiveness of VR-CAP had to be assessed over the long term and beyond the duration of clinical trial follow up. As median survival for VR-CAP had not been reached in the LYM-3002 trial, it was challenging to provide realistic and robust estimates of long-term OS. This challenge is common in UK HTAs and will become more pronounced as regulatory and HTA bodies come under pressure to provide earlier access to promising drugs.

The objective of this study was to assess the cost effectiveness of VR-CAP compared to R-CHOP, in a UK setting, which is currently seen as standard first-line treatment for patients with MCL.

## Methods

### Model structure

**Fig. 1:**
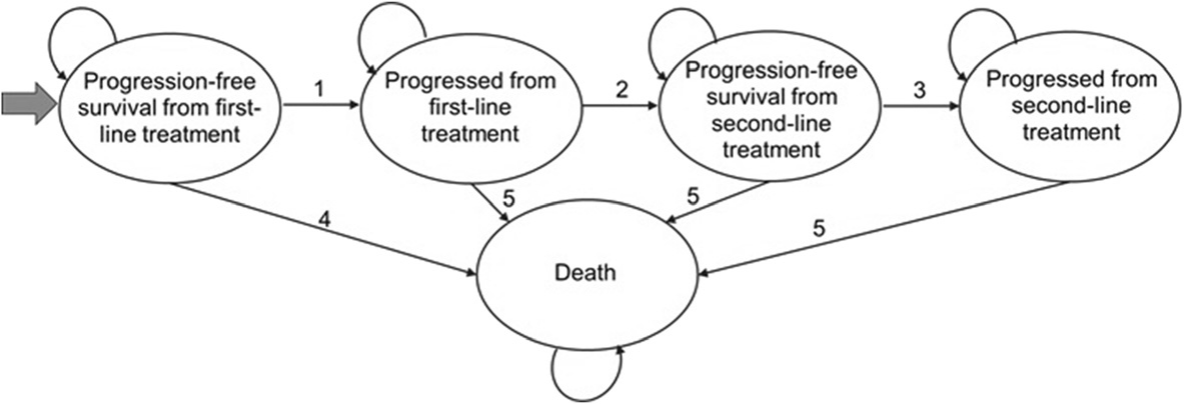
Model diagram. PFS, progression-free survival; PPS, post-progression survival; PrePS, pre-progression survival; TFI, treatment-free interval. 1. Modelled using survival function to PFS Kaplan–Meier data; 2. Modelled using survival function to TFI Kaplan–Meier data; 3. Modelled using average duration of second-line treatment; 4. Modelled using survival function to PrePS Kaplan–Meier curve plus general population background mortality data; 5. Modelled using survival function to PPS Kaplan–Meier curve

The cost-effectiveness model was developed as a Markov model with five health states, representing pre- and post-progression from first- and second-line treatment, as well as death, as presented in Fig. 1. A hypothetical cohort of patients enter the model when they start their first-line treatment for MCL, and their progression through the disease, including second-line treatment, was followed until death. The model used a cycle length of 1 week, at which time patients could move between health states. The cycle length of 1 week was selected to give sufficient granularity to capture short-term changes in progression status. And a lifetime horizon of 20 years was used in line with UK guidance; ≥94 % of patients were modelled to have died within this time horizon [21]. The model used the perspective of the UK National Health Service, and a discount rate of 3.5 % per year for costs and health outcomes as per UK guidance [21].

### Population

The population included in the model was the intention to treat population from the LYM-3002 trial; the only trial investigating the comparative effectiveness of VR-CAP and R-CHOP in MCL (this was confirmed in a systematic literature review). A scenario analysis was performed that included only patients clinically ineligible for HSCT, as LYM-3002 also included patients that were ineligible due to non-clinical reasons (e.g. HSCT was not available or was refused by the patient). Baseline patient characteristics for both populations are presented in Table 1.

**Table 1:** Baseline characteristics of all patients versus non-HSCT eligible patients only in the LYM-3002 trial

Variable	All patients (*n* = 487)	Clinically ineligible for HSCT only (*n* = 407)
Age at baseline	64.29	65.82
Female	26.1 %	26.8 %
European Union	27.9 %	31.2 %
North America	2.9 %	6.3 %
Rest of the World	69.2 %	65.6 %
Stage II	6 %	6 %
Stage III	20 %	22 %
Stage IV	74 %	72 %
ECOG 0	40 %	43 %
ECOG 1	47 %	47 %
ECOG 2	13 %	10 %
Mean patient weight (kg)	70.59	70.03
Body surface area (m^2^)	1.80	1.79

*Abbreviations: ECOG* Eastern Cooperative Oncology Group, *HSCT* haematopoietic stem cell transplantation

### Transitions between health states

**Fig. 2:**
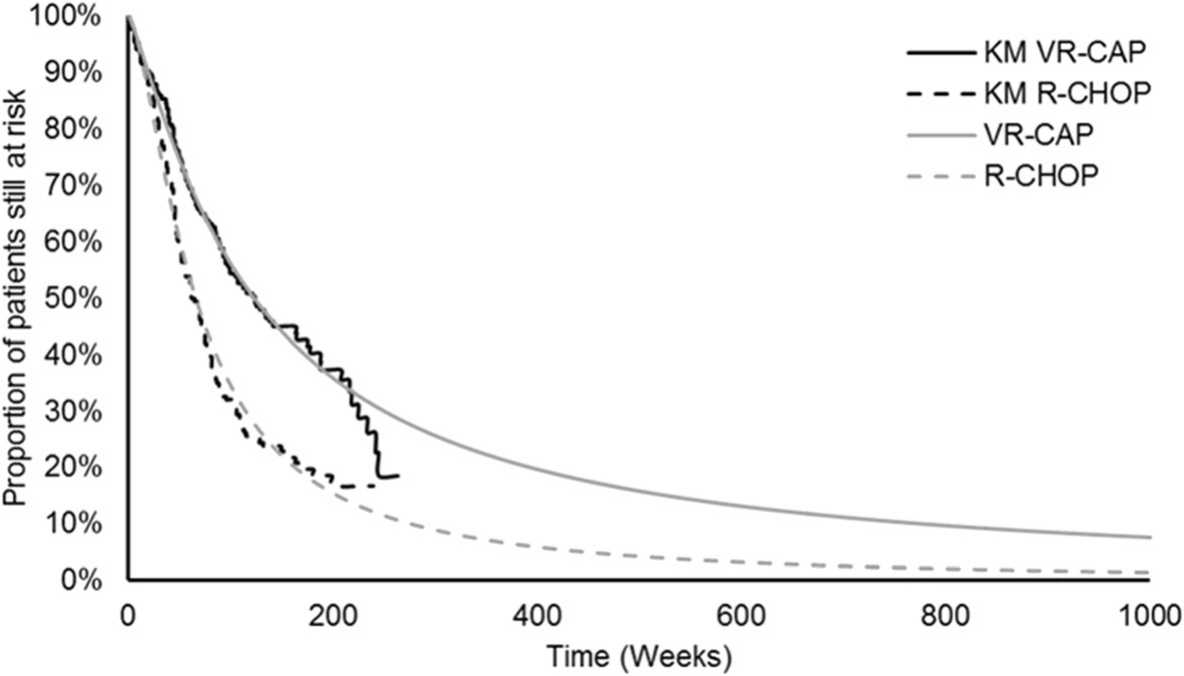
Log-logistic PFS curves used in model base case. KM, Kaplan–Meier; PFS, progression-free survival; R-CHOP, rituximab with cyclophosphamide, doxorubicin, vincristine and prednisolone; VR-CAP, bortezomib with rituximab, cyclophosphamide, doxorubicin and prednisolone

Transitions between health states in the model were based on LYM-3002 data. In addition to PFS by IRC, which was the primary outcome, PFS was also assessed by the investigator and in an alternative IRC assessment. In the primary IRC assessment, patients were classified as progressed when the disease seemed to have worsened based on the International Workshop Response Criteria, on one computerised tomography scan. In the alternative IRC assessment, this could be revised depending on whether a lesion was assessed as resolved or persisting at subsequent time points by the IRC. The alternative IRC assessment of PFS was considered to more closely reflect clinical practice, where more than one scan would be used to assess progression [22]. Scenario analyses were performed to test the impact of the different assessment methods on the model outcomes. To extrapolate beyond the duration of the clinical trial, six different survival functions (exponential, gamma, Gompertz, log-logistic, log-normal and Weibull) were fitted to these PFS trial data, following NICE Decision Support Unit guidance [23]. The choice between survival models was based upon statistical goodness of fit measured using the Akaike information criterion and the Bayesian information criterion (Table 2), visual fit to the trial Kaplan–Meier data, and the validity of the projected survival estimates as assessed by practicing haematologists. The log-logistic model was seen as the most reflective of outcomes observed in clinical practice, and this was therefore used in the model base case (Fig. 2).

**Table 2:** Goodness of fit and model parameters for the PFS, PrePS and PPS curves

		Exponential	Weibull	Log-logistic	Log-Normal	Gamma	Gompertz
PFS VR-CAP	Intercept	7.142	7.146	6.758	6.772	7.148	3.72
	Scale	N/A	1.011	0.839	1.567	1.007	N/A
	Shape	N/A	N/A	N/A	N/A	1.007	−0.0001
	AIC	603.623	605.604	608.385	616.888	607.603	1194.398
	BIC	607.116	612.590	615.371	623.874	618.082	1201.384
PFS R-CHOP	Intercept	6.571	6.566	6.138	6.134	6.374	3.087
	Scale	N/A	0.913	0.654	1.22	1.042	N/A
	Shape	N/A	N/A	N/A	N/A	0.54	−0.005
	AIC	634.079	634.075	622.425	636.948	630.674	1349.269
	BIC	637.576	641.070	629.419	643.942	641.166	1356.263
PrePS and PPS	Intercept	7.61	6.657	7.309	7.355	7.765	4.232
	PrePS VR-CAP	1.511	1.635	1.685	1.979	1.573	1.511
	Pre-PS R-CHOP	1.385	1.499	1.571	1.896	1.412	1.385
	Scale	N/A	1.083	0.964	1.883	1.749	N/A
	Shape	N/A	N/A	N/A	N/A	1.749	0.002
	AIC	915.60	916.37	920.84	929.16	917.11	1717.58
	BIC	932.35	937.31	941.78	950.11	942.24	1738.52

*Abbreviations: AIC* Aikake information criterion, *BIC* Bayesian information criterion, *PFS* progression-free survival, *PPS* post-progression survival, *PrePS* pre-progression survival, *R-CHOP* rituximab with cyclophosphamide, doxorubicin, vincristine and prednisolone, *VR-CAP* bortezomib with rituximab, cyclophosphamide, doxorubicin and prednisolone

Because of the immaturity of OS data, survival functions were stratified by progression status at the end of the trial (pre-progression survival [PrePS] and post-progression survival [PPS]). For non-progressed patients this was also stratified by trial arm. PPS was assumed equal across model arms. This was justified by the observation that PPS was similar for the VR-CAP and R-CHOP arms in the LYM-3002 trial [24], and the expectation that different prior treatments would not be expected to impact PPS [12]. Finally, two studies identified in a literature review of surrogate endpoints in MCL also indicated that PFS may be an appropriate surrogate for OS [25, 26].

**Fig. 3:**
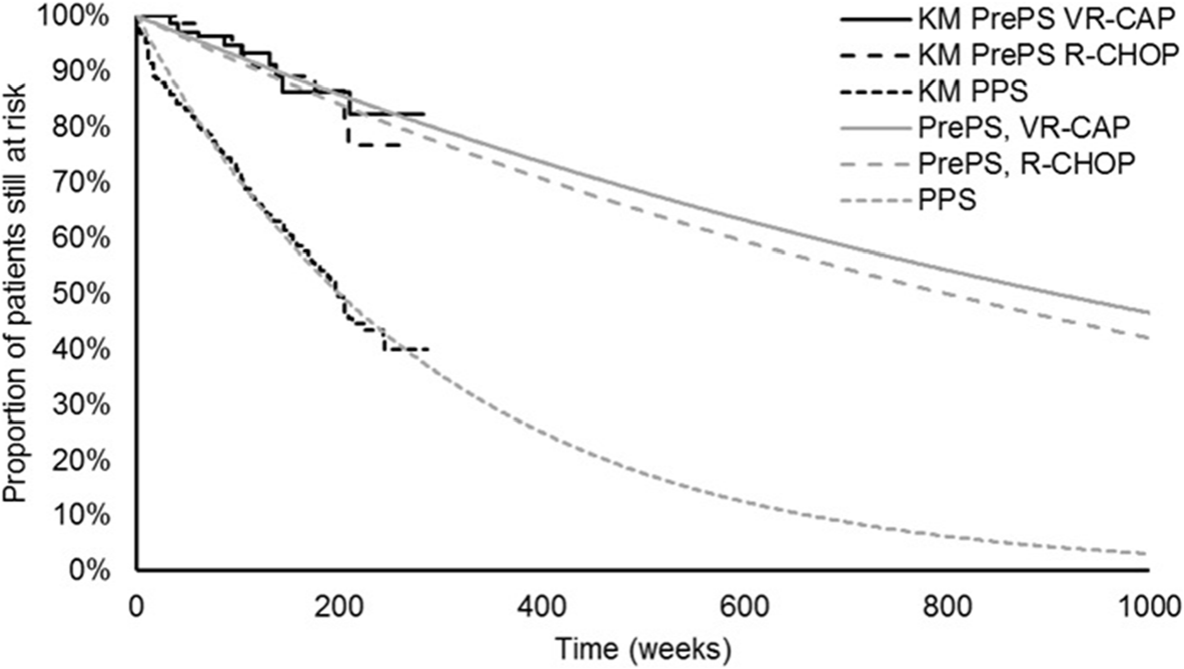
Exponential disease-specific OS curves used in model base case. KM, Kaplan–Meier; OS, overall survival; PrePS, pre-progression survival; PPS, post-progression survival; R-CHOP, rituximab with cyclophosphamide, doxorubicin, vincristine and prednisolone; VR-CAP, bortezomib with rituximab, cyclophosphamide, doxorubicin and prednisolone

Because the long-term projections of PrePS based on extrapolation were quite high, presumably due to the relative immaturity of data, it was decided that non-disease-specific mortality, based on age and gender, should be added to these curves to better capture long-term survival. This was included and based upon UK life tables [27]. For PrePS and PPS, the exponential curves were judged as most reflective of outcomes observed in UK clinical practice (Fig. 3) [12].

**Fig. 4:**
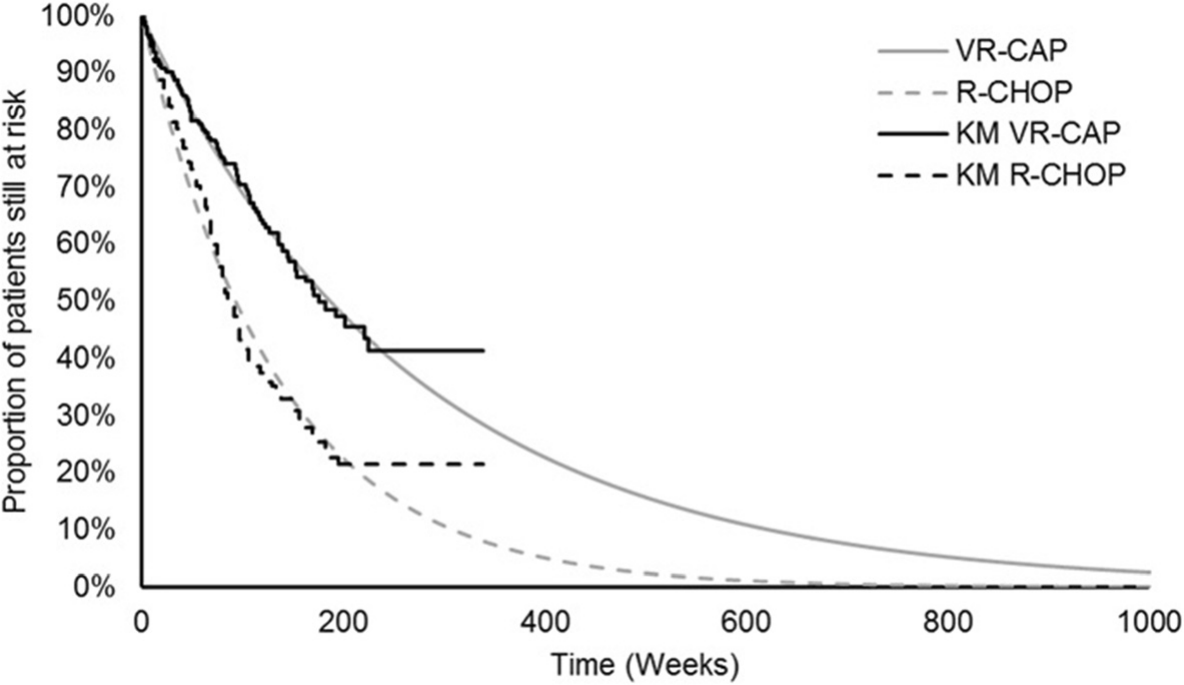
Exponential TFI curves used in model base case. KM, Kaplan–Meier; R-CHOP, rituximab with cyclophosphamide, doxorubicin, vincristine and prednisone; TFI, treatment-free interval; VR-CAP, bortezomib with rituximab, cyclophosphamide, doxorubicin and prednisone

Second-line treatment starts after a treatment-free interval modelled using exponential survival functions (Fig. 4). The distribution of patients over different treatments as well as average duration of treatment (used as a proxy for PFS from second-line treatment; 90 days for both arms) were based on LYM-3002.

### Adverse events

All adverse events (AEs) that happened at Grade 3 or higher in at least 5 % of either treatment group, as well as Grade 2 peripheral sensory neuropathy and Grade 3 or higher alopecia and sepsis, were included in the model, with rates as reported within the LYM-3002 trial. These were selected based on expectation of an important impact on costs, utility or both. The annual rate for each AE was calculated from the number of events in the LYM-3002 trial and the total patient years on treatment. This annual rate was then used to calculate the weekly probability of each AE.

In the model, red blood cell and platelet transfusions were administered to patients to treat AEs and to avoid having to decrease chemotherapy doses. Again, the weekly probability of requiring a transfusion was based on annual rates of administration in LYM-3002 [24].

### Medical resource use and costs

All costs were based on 2013/2014 UK prices. Patient level data from the LYM-3002 trial were used to model the number of patients receiving first-line treatment per treatment cycle. Dose reductions were also applied as they were observed in the trial. Most of the drug doses included in the analysis were based on patient weight or body surface area. To calculate the number of vials required per administration, a distribution was fitted to the patient characteristics observed in the trial. This was then used to calculate the average cost per dose for all patients [28]. Administration costs were applied for all intravenous administrations; for oral drugs one administration visit was assumed at the start of treatment. The use of tests, scans and medical visits was based on advice of UK haematologists and was assumed to vary by treatment status and progression status (Table 3) [24]. Standard UK unit costs were used for treatment, administration, concomitant medication, medical resource use, adverse events and terminal care [29–33]. Treatment, administration and end-of-life costs are summarised in Table 4.

**Table 3:** Medical resource use for disease management by health state (Source of costs: NHS reference costs 2013–2014 [29])

	On treatment (first- or second-line)	Stable disease (off treatment)	At time of progression	Progressed	Unit cost
Full blood count	3 per treatment cycle	1 per 2–3 months^a^	1	0	£3.00
Biochemistry	3 per treatment cycle	1 per 2–3 months^a^	1	0	£1.18
Blood glucose	3 per treatment cycle	0	0	0	£1.18
Computerised tomography scan	In treatment Cycles 1, 3 and 6	0	1	0	£80.00
Haematologist visit	In treatment Cycles 1, 3 and 6	1 per 2–3 months^a^	1	1 per 2–3 months^a^	£150.06

*Abbreviations: NHS* National Health Service

^a^This has been applied to the model as once every 11 weeks

**Table 4:** Cost inputs used in the model

Costs	VR-CAP	R-CHOP	Source
Drug costs per treatment cycle	£4,426	£2,383	MIMS 2015 [31], eMIT 2014 [32]
Administration costs first treatment cycle	£1,116	£381	NHS reference costs 2013–2014 [29]
Administration costs per subsequent treatment cycle	£980	£245	NHS reference costs 2013–2014 [29]
Cost per 90 days of second-line treatment & administration	£11,442	£11,665	MIMS 2015 [31], eMIT 2014 [32]
Cost of care at end of life	£6,018	£6,018	Addicott, 2008 [45], Curtis, 2014 [30]

### Quality of life

Utility scores ranging from 0 to 1, with 0 representing death and 1 representing perfect health, defined the quality of life of patients. In the LYM-3002 trial, utility was measured using the EQ-5D at each cycle of treatment and at the end-of-treatment visit, which was performed 30 days after the last dose was administered. These data were therefore used for the progression-free and progressed from first-line treatment health states. Patients that were progression-free from second-line treatment were assumed to have the same utility as patients progression-free from first-line treatment (Table 5). The economic literature was searched to identify utility values for the progressed from second-line treatment health state; values from aggressive NHL were selected as there were no utilities published specifically for MCL [34]. Decreases in utility for patients experiencing adverse events were also modelled using weekly probabilities of AEs and average durations of AEs from LYM-3002 trial data.

**Table 5:** Utilities applied to the model

Health state	Utility	Source
Progression-free survival from first-line treatment	0.764	LYM-3002 [24]
Progressed from first-line treatment	0.693	LYM-3002 [24]
Progression-free survival from second-line treatment	0.764	LYM-3002 [24]
Progressed from second-line treatment	0.45	Doorduijn, 2005 [34]

### Outcomes

The outcome used in this cost-effectiveness analysis was the cost per quality-adjusted life year (QALY). QALYs were calculated by multiplying the time a patient spent in a specific health state by the utility value associated with that health state. Average lifetime QALYs per patient were calculated as well as average lifetime costs. These were used to calculate the incremental cost-effectiveness ratio (ICER).

### Sensitivity analysis

A series of one-way sensitivity analyses were performed changing one parameter at a time to the upper and lower limit of their 95 % confidence interval, respectively, holding all other parameters constant. This was done to evaluate the sensitivity of the model to individual model inputs. Additionally, a probabilistic sensitivity analysis (PSA) was performed where all parameters at once were randomly sampled from their distribution. This was iterated 1,000 times, so that the uncertainty around the point estimate of the model outcome could be tested. Through empirical testing it was found that 1,000 iterations were sufficient to capture the uncertainty around the base case ICER.

Scenario analyses were also performed testing the assumptions around PFS, OS and utilities, by changing assumptions and using alternative data sources.

### Validation

**Fig. 5:**
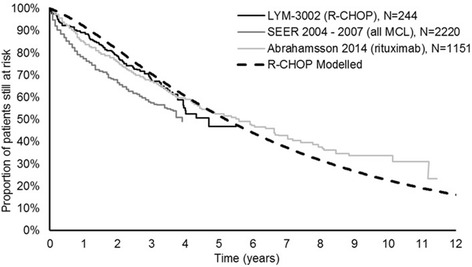
Modelled OS compared to observational datasets. MCL, mantle cell lymphoma; OS, overall survival; SEER, Surveillance, Epidemiology, and End Results Program; R-CHOP, rituximab with cyclophosphamide, doxorubicin, vincristine and prednisone; VR-CAP, bortezomib with rituximab, cyclophosphamide, doxorubicin and prednisone

Because of the uncertainty in the extrapolation of OS data due to immaturity of the data, a comparison of model outcomes to long-term observational studies from inside and outside the UK was made; this showed that outcomes of the model were comparable with contemporaneous long-term datasets (Fig. 5). In comparison to available observational datasets, the survival in the LYM-3002 trial closely followed that reported by Abrahamsson but was greater than that of Surveillance, Epidemiology, and End Results Program (SEER) [35, 36]. Abrahamsson et al. was a recent publication (2014) that reported the OS of a European population (Swedish) and used a similar treatment to the LYM-3002 trial (rituximab-based chemotherapy). In contrast, data from SEER are much older than data from the LYM-3002 trial (2004–2007 versus 2008–2011); the study was conducted in the US and included all MCL treatments (i.e. was likely to include treatments that were less efficacious than R-CHOP).

## Results

**Fig. 6:**
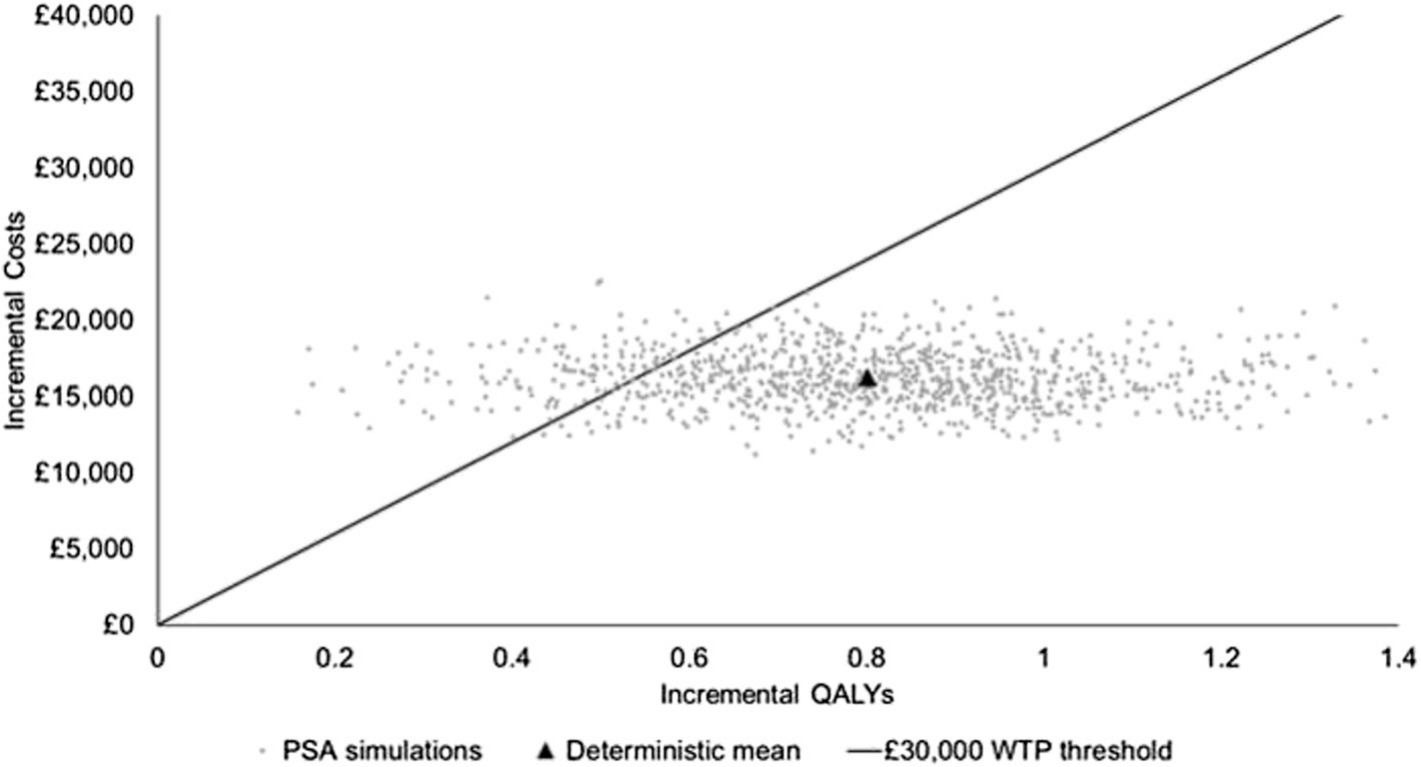
Cost-effectiveness plane from 1,000 PSA iterations. PSA, probabilistic sensitivity analysis; QALY, quality-adjusted life year; WTP, willingness to pay

As presented in Table 6, VR-CAP is associated with higher costs and greater efficacy compared to R-CHOP. The base case results demonstrate that VR-CAP is a cost effective treatment at the conventional UK willingness-to-pay threshold of £20,000–£30,000 per QALY [21] with an ICER of £20,043. The PSA indicated that there was a probability of 88.9 % that the ICER lies below the threshold of £30,000 per QALY. Figure 6 indicates that most uncertainty in the model comes from uncertainty in efficacy.

**Table 6:** Discounted base case model outcomes

	VR-CAP	R-CHOP	Difference^a^
*Deterministic results*			
QALYs	**4.10**	**3.29**	**0.81**
Progression-free survival from first-line treatment	2.70	1.54	1.16
Progressed from first-line treatment	0.14	0.10	0.03
Progression-free survival from second-line treatment	0.12	0.15	−0.03
Progressed from second-line treatment	1.15	1.50	−0.35
Costs	**£45,842**	**£29,630**	**£16,212**
First line therapy medication costs	£22,606	£8,041	£14,566
Administration of first line therapy	£5,817	£1,564	£4,253
Adverse events, transfusions & concomitant medication	£1,472	£1,105	£367
Disease management costs	£4,191	£4,676	−£486
Second line treatment (medication and administration)	£7,152	£9,423	−£2,271
Terminal care	£4,605	£4,821	−£217
Deterministic ICER (£/QALY gained)			**£20,043**
*Probabilistic results*			
QALYs	4.09	3.28	0.81
Costs	£45,482	£29,285	£16,196
Probabilistic ICER (£/QALY gained)			**£19,889**

*Abbreviations: ICER* incremental cost-effectiveness ratio, *QALY* quality-adjusted life year, *R-CHOP* rituximab with cyclophosphamide, doxorubicin, vincristine and prednisolone, *VR-CAP* bortezomib with rituximab, cyclophosphamide, doxorubicin and prednisolone

^a^Some differences due to rounding. First line therapy costs: medication costs of first line treatment; Administration costs: costs of administration of first-line therapies; Adverse events and concomitant medication costs: costs associated with adverse events (treatment of adverse events, concomitant medication and transfusions); Medical resource use; all costs for disease management, such as follow up visits and tests; Second line treatment costs; costs for medication and administration of the subsequent line of treatment; Terminal care costs: costs for end-of-life care

Total costs and QALYs, as well as the ICERs are presented in bold text

Table 6 shows that VR-CAP patients have a longer PFS, whereas R-CHOP patients spend more time in the ‘progressed from second-line treatment’ health state than VR-CAP patients. This is due to the difference in PFS, while PPS is assumed to be equal between arms, generating a smaller difference in OS than PFS. The treatment cost accounts for the majority of the overall costs (Table 6), and therefore uncertainty around resource use and cost sources other than drug costs will have only a minor impact on model outcomes.

**Fig. 7:**
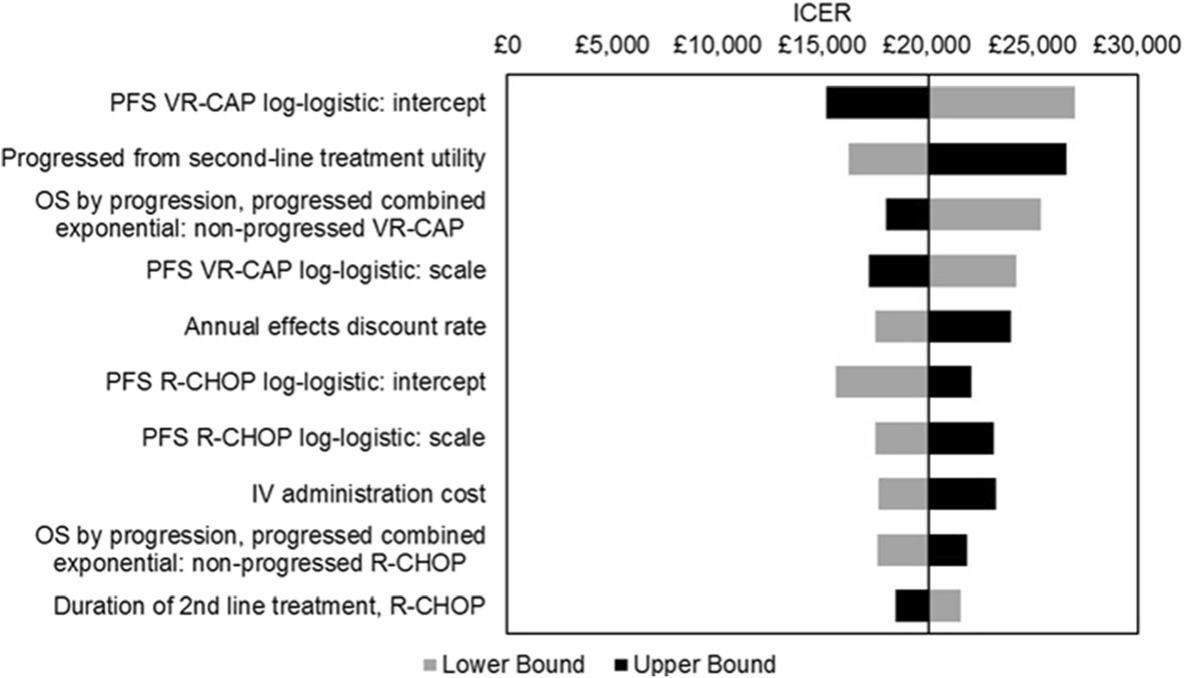
Tornado diagram displaying the ICER sensitivity to the ten most influential model inputs. ICER, incremental cost-effectiveness ratio; IV, intravenous; OS, overall survival; PFS, progression-free survival; R-CHOP, rituximab with cyclophosphamide, doxorubicin, vincristine and prednisone; VR-CAP, bortezomib with rituximab, cyclophosphamide, doxorubicin and prednisone

One-way sensitivity analysis showed that uncertainty in the parameters used within the model for PFS projections had the biggest impact on model outcomes together with the utility value applied to the ‘progressed from second-line treatment’ health state (Fig. 7).

As can be seen from Table 7, the ICER is relatively insensitive to the scenario analyses performed. Using different survival functions for PFS had the largest impact on model outcomes, and alternative sources for utility data for patients progressed from second-line treatment had the largest impact on the ICER. Using different trial assessments of PFS had only a limited impact on outcomes.

**Table 7:** Results of scenario analyses

Scenario	Incremental costs	Incremental QALYs	ICER
Exponential survival function for PFS	£17,366	0.62	£27,789
Weibull survival function for PFS	£17,055	0.64	£25,499
Log-normal survival function for PFS	£15,921	0.77	£18,691
Gamma survival function for PFS	£17,193	0.61	£27,318
Gompertz survival function for PFS	£17,578	0.59	£30,099
Weibull survival function for PrePS and PPS	£16,288	0.72	£20,368
Log-logistic survival function for PrePS and PPS	£16,240	0.57	£22,284
Log-normal survival function for PrePS and PPS	£16,256	0.47	£23,700
Gamma survival function for PrePS and PPS	£16,332	0.81	£19,489
Gompertz survival function for PrePS and PPS	£16,127	0.75	£19,905
Equal PrePS across arms	£16,015	0.68	£20,639
OS by trial arm instead of PrePS and PPS	£15,536	0.73	£21,357
Primary IRC assessment of PFS	£16,009	0.65	£21,369
Investigator assessment of PFS	£16,586	0.91	£18,737
Patients clinically ineligible for HSCT only	£16,257	0.79	£20,195
All utilities based on Doorduijn 2005 [34]	£16,212	0.75	£28,419
Using utility decrement for progressing based on Doorduijn 2005 [34]	£16,212	0.69	£23,409

*Abbreviations: HSCT* hematopoietic stem cell transplantation, *ICER* incremental cost-effectiveness ratio, *IRC* independent review committee, *OS* overall survival, *PFS* progression-free survival, *PPS* post-progression survival, *PrePS* pre-progression survival, *QALY* quality-adjusted life year

## Discussion

The base case ICER of £20,043 indicates that VR-CAP is a cost-effective treatment option for patients with previously untreated MCL, using the standard UK threshold of £20,000–30,000 per QALY.

In the analysis, PFS is used as a surrogate for OS. This approach assumes that there is no survival benefit after a patients disease has progressed following treatment. When OS data were used directly to model cost effectiveness, the ICER increased slightly to £21,357. In this scenario it is assumed that there is a continued benefit of VR-CAP over R-CHOP after disease progression. The observation that OS, as modelled in the base case, shows a good reflection of the LYM-3002 data supports the use of PFS as a surrogate in the base case. A targeted literature review of NICE appraisals for cancer drugs from 2010 onwards identified two recent examples where PFS was used as a surrogate for OS either directly or indirectly (by assuming the same post-progression survival [PPS]) [37, 38]. In both cases, this methodology came under substantial scrutiny. Additionally, three submissions were identified where the same PPS was applied for all treatment arms [39–41].

There are some differences between the LYM-3002 trial population and MCL patients in the UK. As is often the case in clinical trials, the mean age of participants in LYM-3002 (64 years) was relatively low, compared with most patients who present at a median age of 73.5 in clinical practice in the UK [42]. Additionally, only 30 % of patients enrolled in LYM-3002 came from the European Union or North America, with no patients included from the UK. However, efficacy results showed consistency between geographic regions both in the size of benefit with VR-CAP and the absolute PFS for R-CHOP. It is therefore unlikely that the geographic spread of countries included in the trial and the lack of UK patients had any relevant impact upon the results.

The status of the OS data is the main uncertainty in assessing the cost effectiveness of treatment. Despite the conclusion that modelled OS was reasonably comparable to long-term datasets, OS data for VR-CAP are immature. Once the final analysis of OS for LYM-3002 is available, the model could be re-assessed to confirm robustness of the current analysis.

The model does not take into account rituximab maintenance (R-maintenance) treatment for patients that respond to induction therapy, which has been adopted in clinical practice in recent years based on the findings of the European MCL Elderly trial [11]. At the time of initiation of LYM-3002, R-maintenance was not commonly adopted and thus was not included in the trial design. There is a believe that R-maintenance therapy results in similar benefit after any CHOP-like induction regimen, and therefore we would expect to be able to give R-maintenance after VR-CAP induction with a similar extension to median survival times as observed with R-maintenance after R-CHOP induction [43]. As the European MCL Elderly trial was not designed to assess the clinical efficacy of induction therapy with versus without maintenance therapy, it could not be used to model R-maintenance.

When submitted to NICE, the evidence review group agreed that immature data may bias the extrapolation of survival data, and had some concerns about the methods used to overcome this. It was argued that if data are too immature to model OS for all patients, it would be questionable whether sufficient data are available to separately estimate long-term survival for patients with and without progression. However, the uncertainty was reduced for patients who had progressed as a smaller proportion of patients at risk were still alive at the time of evaluation. Furthermore, the data for the two treatment arms is pooled and thereby the total sample size is increased. The uncertainty of survival for patients who had not progressed may be increased by using this method, but this was accounted for by including general population mortality for patients that had not yet progressed. In doing so, it was assumed that all deaths in the PrePS curves (prior to adjustment for background mortality) in the trial were deaths from MCL. This was a reasonable assumption as the number of deaths reported in the LYM-3002 trial that were not due to progression or toxicity was very low. Of the 69 deaths in total in the VR-CAP group, there were only eight deaths that were not due to progression or AEs. In the R-CHOP group, there were a total of 87 deaths, of which 14 were not due to progression or AEs [22].

A submission for HTA was also made to the Scottish Medicines Consortium (SMC), who also noted that there are limitations arising from the maturity of the survival data, but found it unlikely that the approach taken would cause substantial bias in favour of VR-CAP. The SMC noted that this was supported by the literature providing evidence of an association between PFS and OS in MCL. In addition, it was noted that the modest impact on the ICER from uncertainty associated with varying survival inputs meant that the ICER for VR-CAP was robust [44].

In 2015 both NICE and the SMC accepted the overall approach taken in the cost-effectiveness model as a basis for their conclusion that VR-CAP represents a cost-effective treatment option for previously untreated MCL for whom HSCT is unsuitable, in the UK [22, 44]. VR-CAP is now recommended for use within the National Health Service.

## Conclusion

The current model shows that VR-CAP is a cost effective treatment option for patients with previously untreated MCL, for whom haematopoietic stem cell transplantation is unsuitable, in the UK. Both NICE and SMC have recommended the use of VR-CAP in these patients.

## Abbreviations

AE, adverse event; ECOG, Eastern Cooperative Oncology Group; HMRN, Haematological Malignancy Research Network; HSCT, haematopoietic stem cell transplantation; HTA, health technology assessment; ICER, incremental cost-effectiveness ratio; IRC, independent review committee; IV, intravenous; MCL, mantle cell lymphoma; NHL, non-hodgkin lymphoma; NHS, National Health Services; NICE, National Institute for Health and Care Excellence; OS, overall survival; PFS, progression-free survival; PPS, post-progression survival; PrePS, pre-progression survival; PSA, probabilistic sensitivity analysis; QALY, quality-adjusted life year; R, rituximab; R-CHOP, rituximab, cyclophosphamide, doxorubicin, vincristine and prednisolone; R-FC, rituximab, fludarabine and cyclophosphamide; SEER, Surveillance, Epidemiology, and End Results Program; SMC, Scottish Medicines Consortium; TFI, treatment-free interval; UK, United Kingdom; VR-CAP, bortezomib, rituximab, cyclophosphamide, doxorubicin and prednisolone

## Additional file

Additional file 1: List of local Independent Ethics Committees and Institutional Review Boards. (DOCX 24 kb)
