# Supplementary material for: Cost-effectiveness analysis of bortezomib in combination with rituximab, cyclophosphamide, doxorubicin, vincristine and prednisone (VR-CAP) in patients with previously untreated mantle cell lymphoma
Source: BMC Cancer. 2016 Aug 4;16(1):598. doi: 10.1186/s12885-016-2633-2 (PMC4972997; doi:10.1186/s12885-016-2633-2)
Supplement: Additional file 1: — List of local Independent Ethics Committees and Institutional Review Boards. (DOCX 24 kb) [file 12885_2016_2633_MOESM1_ESM.docx]

| **Country** | **Study site** | **Name of Institutional Review Board/Ethics Committee** |
| --- | --- | --- |
| Austria | Allgemeines Krankenhaus der Stadt Wien | Ethikkommission d. Med. Universität Wien und des Allgemeinen Krankenhauses der Stadt Wien |
|  | St.Johanns Spital/Landeskrankenhaus Salzburg | Ethikkommission für das Bundesland Salzburg Amt der Salzburger Landesregierun |
|  | Univ.Klinik f. Innere Medizin Innsbruck | Ethikkommission der Medizinischen Universität Innsbruck Geschäftsstelle der Ethikommission |
| Belgium | Universitair Ziekenhuis Gent | Ethisch Comité Universitair Ziekenhuis Gent |
|  | UZ Leuven Gasthuisberg | COMISSIE MEDISCHE ETHIEK |
|  | UZ Brussel | Commissie Medische Ethiek AZ VUB |
|  | UZA Antwerpen | ETHICS COMMITTEE UNIVERSITY HOSPITAL ANTWERP |
|  | AZ Stuivenberg Antwerpen | Commissie voor Medische Ethiek - ZNA/OCMW Antwerpen |
|  | UCL DE MONT-GODINNE Yvoir | COMITE D ETHIQUE CLINIQUES UNIVERSITAIRES DE MONT GODINNE |
|  | C.H.R. Citadelle, Liège | Comité d'ethique CHR La Citadelle |
|  | CHU SART TILMAN, Liège | Comité d'ethique CHU SART-TILMAN |
|  | AZ ST. JAN, Brugge | Commissie Medische Ethiek AZ Sint Jan Brugge |
| Brazil | CENTRO DE ESTUDOS DE HEMATOLOGIA E ONCOLOGIA DA FMABC, Santo Andre | CEP - Faculdade de Medicina do ABC |
|  | INCA - INSTITUTO NACIONAL DO CANCÊR, Rio De Janeiro | CEP - INCA |
|  | HOSPITAL SAO LUCAS PUC-RS, Porto Alegre | CEP - PUC RIO GRANDE DO SUL |
|  | Hospital das Clínicas da Faculdade de Medicina da USP, Sao Paulo | CEP - Hospital das Clinicas da FMUSP |
|  | Fundacao Pio XII - Hospital de Cancer de Barettos | CEP - FUND PIO XII - HOSP CANCER DE BARRETOS |
|  | Hospital Nossa Senhora da Conceicao, Porto Alegre | CEP - Hospital Nossa Senhora da Conceicao |
|  | FUNDACAO HOSPITAL AMARAL CARVALHO, Jau | CEP - FUNDACAO HOSPITAL AMARAL CARVALHO |
|  | HOSPITAL ALEMAO OSWALDO CRUZ R, Sao Paulo | CEP - HOSPITAL ALEMAO OSWALDO CRUZ |
|  | CENTRO DE HEMATOLOGIA E HEMOTERAPIA - UNICAMP, Campinas | CEP - FACULDADE DE CIENCIAS MEDICAS - UNICAMP CIDADE UNIVERSITARIA ZEFERINO VAZ CAMPINAS |
|  | SANTA CASA DE MISERICÓRIDA DE SÃO PAULO | CEP - Santa Casa de Misericordia de Sao Paulo |
|  | HOSPITAL AC CAMARGO, Sao Paulo | CEP - Hospital do Cancer - AC Camargo |
| Canada | Cross Cancer Institute, Edmonton | Alberta Health Services |
|  | Princess Margaret Hospital - University, Toronto | Ontario Cancer Research Ethics Board |
| Chile | INSTITUTO NACIONAL DEL CANCER, Santiago | Comité de Ética de la Investigación - Servicio de Salud Metropolitano Norte |
|  | HOSPITAL DEL SALVADOR, Providencia | COMITÉ DE ÉTICA CIENTÍFICO DEL SERVICIO DE SALUD METROPOLITANO ORIENTE COMITE ETICO CIENTIFICO DEL SSM CENTRAL |
|  | Hospital Base Valdivia | Comité Ética de Investigación - Servicio de Salud Valdivia |
| China | Cancer Institute & Cancer Hospital, Beijing | NCC Ethics Committee/IRB |
|  | Beijing Cancer Hospital | Beijing Cancer Hospital IEC |
|  | Peking University Third Hospital | Drug Clinical Research IRB of Peking University Third Hospital |
|  | Tianjin Medical University Cancer Hospital and Institute | Tianjin Medical University Cancer Hospital and Institute |
|  | Ruijin Hospital, Shanghai | EC of Rui Jin Hospital |
|  | Cancer hospital,MCFDU, Shanghai | EC of Cancer Hospital, Fudan University |
|  | Sun Yat-sen University Cancer Center, Guangzhou | Sun Yat-sen University Oncology Center |
|  | Zhejiang University First Hospital, Unk Hangzhou | The Frist Affiliated Hospital of Zhejiang University of IRB/EC |
|  | West China Hospital, Sichuan University | EC of West China Hospital |
|  | Beijing Friendship Hospital | EC of Beijing Friendship Hospital |
| Colombia | Fundacion*, Medellin | COMITE DE ETICA DE INVESTIGACION DE LA ORGANIZACION SANITAS INTERNACIONAL |
|  | FUNDACION UNIVERSITARIA SANITAS- CONSULTORIO DR CARLOS ALBERTO RAMIREZ CERON, Bogota | COMITE DE ETICA DE INVESTIGACION DE LA ORGANIZACION SANITAS INTERNACIONAL |
|  | HOSPITAL PABLO TOBÓN URIBE, Medellin | COMITE DE INVESTIGACIONES Y ETICA EN INVESTIGACION- HPTU |
|  | CIOSAD S.A., Bogota | COMITE DE ETICA - INVESTIGACION CLINICA CON SERES HUMANOS |
| Czech Republic | Internal Haematooncology Department FN Brno | Eticka komise Fakultni nemocnice Brno |
|  | Department of Clinical Haematology FN HK, Hradec Kralove | Eticka komise, FN Hradec Kralove |
|  | Department of Clinical Hematology FNKV, Praha | Eticka komise fakultni nemocnice Kralovske Vinohrady |
|  | Ustav klinicke hematologie, Ostrava | Lokalni Eticka komise FN Ostrava |
| France | Hôpital Albert Michallon, Grenoble | CPP SUD EST V |
|  | Service Hématologie, Nantes | CPP SUD EST V |
|  | Centre Hospitalier Emile Muller, Mulhouse | CPP SUD EST V |
|  | Institut Bergonié, Centre de Lutte Contre le Cancer, Bordeaux | CPP SUD EST V |
| Germany | Tumorklinik Sanafontis, Freiburg | ETHIKKOMMISSION BEI DER LANDESÄRZTEKAMMER BADEN-WÜRTTEMBERG |
|  | Vivantes Klinikum Spandau - 2. Klinik für Innere Medizin, Berlin | Landesamt für Gesundheit und Soziales Berlin Geschaeftsstelle der Ethik-Kommission des Landes Berlin |
|  | Vivantes Klinikum Neukölln, Berlin | Landesamt für Gesundheit und Soziales Berlin Geschaeftsstelle der Ethik-Kommission des Landes Berlin |
|  | Schwarzwald-Baar-Klinikum Villingen- Schwenningen GmbH / Klinik für Innere Medizin II - Germany | ETHIKKOMMISSION BEI DER LANDESÄRZTEKAMMER BADEN-WÜRTTEMBERG JAHNSTRASSE 40 |
|  | Klinikum Frankfurt Höchst GmbH | ETHIKKOMMISSION DER LANDESARZTEKAMMER HESSEN |
|  | Johannes-Gutenberg-Universität, III Med. Klinik | Ethik-Kommission der Landesaerztekammer Rheinland-Pfalz |
|  | Klinikum Lippe-Lemgo GmbH; Med. Klinik II- Germany | ETHIKKOMMISSION DER ÄRZTEKAMMER WESTFALEN-LIPPE UND DER MED. FAKULTÄT DER WWU-MÜNSTER |
|  | Evang. Krankenhaus Hamm gGmbh- Hämatologie/Onkologie | Ethikkommission ETHIKKOMMISSION DER ÄRZTEKAMMER WESTFALEN-LIPPE UND DER MED. FAKULTÄT DER WWU-MÜNSTER |
|  | Universitaetsklinikum Frankfurt | Ethik-Kommission des Fachbereichs Medizin der Johann-Wolfgang-Goethe Universität |
|  | Robert-Bosch-Krankenhaus - Hämatologie / Onkologie | ETHIKKOMMISSION BEI DER LANDESÄRZTEKAMMER BADEN-WÜRTTEMBERG |
|  | Onkologische Schwerpunktpraxis Dr. Kasper | Ethik-Kommission der Bayerischen Landesarztkammer |
|  | Klinikum Chemnitz GmbH, Klinik für Innere Medizin | Ethikkommission bei der Saechsischen Landesaerztekammer |
|  | Caritas Krankenhaus, Lebach | Ethikkommission bei der Aerztekammer der Saarlandes |
|  | MediProjekt - Germany, Hannover | Ethikkommission der Ärztekammer Niedersachsen; Unterkomm. zur Begutacht. med. Forschung am Menschen Körperschaft Des Öffentlichen Rechst |
|  | Gemeinschaftsparaxis Dr. Lerchenmüller/Dr. Kratz-Albers - Germany | ETHIKKOMMISSION DER ÄRZTEKAMMER WESTFALEN-LIPPE UND DER MED. FAKULTÄT DER WWU-MÜNSTER |
| Hungary | KAPOSI MOR MEGYEI KORHAZ KAPOSVAR | Medical Research Council Hungary |
|  | PETZ ALADAR MEGYEI OKTATO KORHAZ, UROLOGIA | Medical Research Council Hungary |
|  | Debreceni Egyetem Orvos es Egeszsegtudomanyi Centrum Nagyerdei krt. 98 | Medical Research Council Hungary |
|  | Szegedi Tudományegyetem | Medical Research Council Hungary |
|  | Semmelweis Egyetem I.Belgyogyaszati Klinika | Medical Research Council Hungary |
|  | Debreceni Egyetem Orvos- és Egészségtudományi Centrum | Medical Research Council Hungary |
| India | Regional Cancer Centre, Trivandium | Human Ethics Committee |
|  | Kidwai Memorial Institute of Oncology | The Medical Ethics Committee |
|  | Jehangir Clinical Development Center Pvt Ltd | Hirabai Cowasji Jehangir |
|  | N S Bose Cancer Research Institute | Ethics Committee |
| Israel | Rabin Medical Center, Beilinson Campus | Helsinki Committee - Rabin |
|  | Sheba Medical Center, Ramat-Gan | Helsinki Committee - Chaim |
|  | Kaplan Medical Center - Hematology | Kaplan Helsinki Comittee |
|  | Hadassah Hospital | Helsinki Committee - Hadassah |
|  | RAMBAM MEDICAL CENTER Bat Galim | Helsinki Committee - Rambam |
|  | BNEI ZION MEDICAL CENTER Haifa | Bnai Zion Helsinki Comittee |
| Italy | Istituto di Ematologia Seràgnoli azienda ospedaliera univeristaria Policlinico S.Orsola-Malpighi | COMITATO ETICO INDIPENDENTE DELL'AZIENDA OSPEDALIERO- UNIVERSITARIAPOLICLINI CO S. ORSOLA-MALPIGHI |
|  | Sezione Autonoma Ematologia - Spedali | COMITATO ETICO AZIENDA SPEDALI CIVILI DI BRESCIA |
|  | SC EMATOLOGIA, Torino | Comitato Etico dell'Azienda Ospedaliera San Giovanni Battista |
|  | DIPARTIMENTO DI ONCOLOGIA ED EMATOLOGIA, Modena | COMITATO ETICO DELLA PROVINCIA DI MODENA |
|  | AMADORI SERGIO, Roma | Comitato Etico Azienda Ospedaliero Universitaria Policlincico Tor Vergata |
|  | Policlinico Le Scotte, Sienna | COMITATO ETICO LOCALE PER LA SPERIMENTAZIONE CLINICA DEI MEDICINALI DELL`AZIENDA OSPEDALIERA UNIVERS |
|  | OSPEDALE CA GRANDA - NIGUARDA, Milano | COMITATO ETICO LOCALE PER LA SPERIMENTAZIONE CLINICA DELL'OSPEDALE LUIGI SACCO DI MILANO |
|  | Centro di Riferimento Oncologico di Basilicata, Rionero in Vulture | Comitato Etico CROB |
| Japan | National Hospital, Hokkaido Cancer Center, Sapporo | National Hospital Organization Hokkaido Cancer Center Institutional Review Board |
|  | Tohoku University Hospital, Sendai | Tohoku University Hospital Institutional Review Board |
|  | Tokyo Metropolitan - Komagome Hospital | Tokyo Metropolitan Cancer and Infectious diseases Center Komagome Hospital IRB/IEC |
|  | National Cancer Center Central Hospital | The Institutional Review Board of National Cancer Center |
|  | National Cancer Center Hospital East | The Institutional Review Board of National Cancer Center |
|  | The Cancer Institute Hospital of JFCR | The Institute Hospital of JFCR Institutional Review Board |
|  | Tokai University Hospital | Tokai University Institutions Review Board |
|  | Nagoya Second Red Cross Hospital | Nagoya Daini Red Cross Hospital IRB/IEC |
|  | Nagoya University Hospital | Nagoya University Hospital IRB |
|  | Kyoto Prefectural University of Medicine | Kyoto Prefectural University of Medicine IRB |
|  | National Kyushu Cancer Center | National Kyushu Cancer Center Institutional Review Board |
| Korea | Asan Medical Center | IRB of Asan medical center |
|  | Korea Cancer Center Hospital | Korea Cancer Center Hospital IRB |
|  | Kyungpook National University Hospital | Kyungpook National University Hospital IRB |
|  | Samsung Medical Center - Dept.of Internal Medicine | Samsung Medical Center IRB |
| Malaysia | University Malaya Medical Centre | Medical Ethics Committee University Malaya Medical Centre |
| Morocco | Centre d'oncologie Al Azhar Centre D'oncologie Al Azhar Rabat | Comité d'éthique pour la recherche biomédicale Faculté de médecine et de Pharmacie Département de Pharmacologie |
|  | Hopital Du 20 Aout 1953 | Comité d'éthique pour la recherche biomédicale Faculté de médecine et de Pharmacie Département de Pharmacologie |
|  | Institut National d Oncologie Sidi Mohamed | Comité d'éthique pour la recherche biomédicale Faculté de médecine et de Pharmacie Département de Pharmacologie 19 rue Tarik Bnou Ziad |
| Philippines | St Luke's Medical Center | St Luke's Medical Center |
|  | National Kidney and Transplant Institute | National Kidney and Transplant Institute |
| Poland | Klinika Hematologii AM, Lodz | Komisja Bioetyki Uniwersytet Medyczny w Lodzi |
|  | Klinika Hematologii AM, Wroclaw | Komisja Bioetyki Uniwersytet Medyczny w Lodzi |
|  | Szpital Morski, Oddzial Chemioterapii | Komisja Bioetyki Uniwersytet Medyczny w Lodzi |
|  | Szpitali Miejskich Bielawskiego Chorzow | Komisja Bioetyki Uniwersytet Medyczny w Lodzi |
| Portugal | HOSPITAL DE SANTA MARIA AV | Comissao de Etica para a Investigacao Clinica |
|  | Hospitais da Universidade de Coimbra | Comissao de Etica para a Investigacao Clinica |
|  | Instituto Portugues de Oncologia | Comissao de Etica para a Investigacao Clinica |
|  | Hospital de Braga | Comissao de Etica para a Investigacao Clinica |
|  | Hospital Geral de Santo Antonio | Comissao de Etica para a Investigacao Clinica |
| Romania | TRUICA CRISTINA, M.D., Baia Mare | Central Ethics Committee Romania |
|  | Spitalul Universitar de Urgenta, Sectia Hematologie | Central Ethics Committee Romania |
|  | Institutul Clinic Fundeni, Clinica de Hematologie | Central Ethics Committee Romania |
|  | BURCOVEANU Cristina, Iasi | Central Ethics Committee Romania |
|  | Cebotaru Cr., Cluj-Napoca | Central Ethics Committee Romania |
| Russia | S.P. Botkin Moscow City Clinical Hospital | Independent Interdisciplinary Committee for Ethics Expertise of Clinical Trials |
|  | St.-Petersburg City Clinical Hospital nr 31 | Independent Interdisciplinary Committee for Ethics Expertise of Clinical Trials |
|  | Leningrad Region Clinical Hospital | Independent Interdisciplinary Committee for Ethics Expertise of Clinical Trials |
|  | CANCER RESEARCH CENTER RAMS - N.N. BLOKHIN - ACADEMY OF MEDICAL SCIENCE | Independent Interdisciplinary Committee for Ethics Expertise of Clinical Trials |
|  | Chelyabinsk Regiona Onc. Center | Independent Interdisciplinary Committee for Ethics Expertise of Clinical Trials |
|  | St.-Petersburg Clinical Research Institute of Hematology and Transfusiology | Independent Interdisciplinary Committee for Ethics Expertise of Clinical Trials |
|  | Moscow Regional Clinical Research Institute | Independent Interdisciplinary Committee for Ethics Expertise of Clinical Trials |
|  | Medical Scientific Radiology - Center | Independent Interdisciplinary Committee for Ethics Expertise of Clinical Trials |
|  | Omsk Regional Oncology Dispensary | Independent Interdisciplinary Committee for Ethics Expertise of Clinical Trilas |
|  | Nizhniy Novgorod Region Clinical Hospital | Independent Interdisciplinary Committee for Ethics Expertise of Clinical Trilas |
|  | Novosibirsk Region Clinical Hospital | Independent Interdisciplinary Committee for Ethics Expertise of Clinical Trilas |
|  | 1st Republican Clinical Hospital of Udmurtia | Independent Interdisciplinary Committee for Ethics Expertise of Clinical Trilas |
|  | Sverdlovsk Regional Oncology Dispensary | Independent Interdisciplinary Committee for Ethics Expertise of Clinical Trilas |
|  | Perm Medical Sanitary Unit#1 | Independent Interdisciplinary Committee for Ethics Expertise of Clinical Trilas |
|  | Belgorod Regional Oncology Center | Independent Interdisciplinary Committee for Ethics Expertise of Clinical Trilas |
|  | City Clinical Oncology Dispensary | Independent Interdisciplinary Committee for Ethics Expertise of Clinical Trilas |
|  | Rostov Research Institute of Oncology | Independent Interdisciplinary Committee for Ethics Expertise of Clinical Trilas |
|  | Arkhangelsk Regional Clinical Hospital | Independent Interdisciplinary Committee for Ethics Expertise of Clinical Trilas |
|  | Hematology Scientific Center | Independent Interdisciplinary Committee for Ethics Expertise of Clinical Trilas |
|  | Federal Center of Heart, Blood and Endocrinology | Independent Interdisciplinary Committee for Ethics Expertise of Clinical Trilas |
|  | Clinical Oncology Center of Tatarstan | Independent Interdisciplinary Committee for Ethics Expertise of Clinical Trilas |
|  | Sochi Oncology Dispensary | Independent Interdisciplinary Committee for Ethics Expertise of Clinical Trilas |
|  | Clinical oncology dispenser 1 of Krasnodar region | Independent Interdisciplinary Committee for Ethics Expertise of Clinical Trilas |
|  | Singapore General Hospital - Hematology | Review Board Singhealth Centralized Institutional Review Board |
| Singapore | National Cancer Centre | Centralized Independent Review Board Singhealth Centralized Institutional Review Board |
| South Africa | Medical Oncology Center of Rosebank | Pharma-Ethics |
|  | Drs Pirjol & Szpak inco-operated | Pharma-Ethics |
|  | Wits Donald Gordon Clinical Trial Site 18 | Wits Health Consortium |
| Spain | HOSPITAL CLINIC I PROVINCIAL DE BARCELONA | HOSPITAL CLINIC I PROVINCIAL COMITE ETICO DE INVESTIGACION CLINICA |
|  | Hospital Universitario de La Princesa | HOSPITAL LA PRINCESA - COMITE ETICO DE INVESTIGACION CLINICA |
|  | Hospital Clinico Universitario Salamanca | EC OF HOSPITAL UNIVERSITARIO DE SALAMANCA-UNIDAD DE INFECCIOSAS |
|  | Hospital Universitario Germans Trias i pujol Edificio Maternal Carretera De Canyet Badalona | Secretaria i Coordinació Comitè Ètic d'Investigació Clínica del Hospital Germans Trias i Pujol |
|  | GONZALEZ-BARCA EVA, L'hospitalet de Llobregat | CEIC HU DE BELLVITGE - SECRETARÍA ADMINISTRATIVA - UNITAT DE SUPORT A LA RECERCA |
|  | Hospital 12 de Octubre | CEIC HOSPITAL 12 DE OCTUBRE |
|  | Hospital Donostia | HOSPITAL DE DONOSTIA - SECRETARIA CEIC |
|  | Hospital Puerta del Mar | CEIC HOSPITAL UNIVERSITARIO PUERTA DEL MAR |
|  | Hospital Clínico San Carlos | HOSPITAL CLINICO SAN CARLOS COMITE ETICO DE INVESTIGACION CLINICA |
| Taiwan | Chang-Gung Memorial Hospital, Linkou | Chang-Gung Memorial Hospital, Linkou |
| Thailand | Ramathibodi Hospital | COMMITTEE ON HUMAN RIGHTS RAMATHIBODI HOSPITAL |
|  | Siriraj Hospital-Hematology Unit | Research Ethics Siriraj Hospital |
|  | Maharaj Nakorn Chiang Mai hospital - Faculty of Medicine | Research Ethics Chiang Mai |
|  | King Chulalongkorn Memorial Hospital Division Of Hematology Department Of Medicine | The Ethics Committee Faculty of Medicine Chulalongkorn |
| Tunisia | Centre National de Greffe de Moelle osseuse | Comité d'éthique et de protection des personnes E P S Hopital charles Nicolle |
|  | Hôpital Farhat Hached | Comité d'éthique et de recherche Hôpital Farhat Hached Avenue |
|  | Hôpital Aziza Othmana | Comité d'éthique Hopital La Rabta |
|  | Institut Salah Azaiz | Comité déthique Institut Salah AZAIEZ |
| Turkey | DOKUZ EYLUL UNIVERSITY MED. FAC. | DOKUZ EYLUL UNIVERSITY MED. FAC.  Istanbul University Istanbul Medical Faculty Clinical Research Ethics Committee |
|  | HACETTEPE UNIVERSITY MEDICAL FACULTY | DOKUZ EYLUL UNIVERSITY MED. FAC.  Istanbul University Istanbul Medical Faculty Clinical Research Ethics Committee |
|  | Dr. Lutfi Kirdar Training and Research | DOKUZ EYLUL UNIVERSITY MED. FAC.  Istanbul University Istanbul Medical Faculty Clinical Research Ethics Committee |
|  | Erciyes University Medical Faculty Dept. of Hematology | Istanbul University Istanbul Medical Faculty Clinical Research Ethics Committee |
|  | On Dokuz Mayis University Medical | Istanbul University Istanbul Medical Faculty Clinical Research Ethics Committee |
|  | Gaziantep University Medical Faculty Dept. of Psychiatry | Istanbul University Istanbul Medical Faculty Clinical Research Ethics Committee |
| Ukraine | National Cancer Institute, Dept. of chemotherapy of hemoblastosis | Central Ethics Commission of MoH of Ukraine Local Ethic Committee of National Cancer Institute |
|  | Crimean Republic Clinical Oncology | Central Ethics Commission of MoH of Ukraine Local Ethic Committee of National Cancer Institute |
|  | Dispensary, Haematology Department | Central Ethics Commission of MoH of Ukraine Local Ethic Committee of Crimean Republic Clinical Oncology Dispensary |
|  | Khmelnitskiy Regional Hospital, Hematology Department | Central Ethics Commission of MoH of Ukraine Ethics Commission of Khmelnitskiy Regional Hospital |
|  | Institute of Blood Pathology and Transfusion Medicine, Lviv Clinical Hospital #5, Hematology Dept. | Central Ethics Commission of MoH of Ukraine Ethics Commission of Institute of blood pathology and transfusion medicine of AMS of Ukraine |
|  | Cherkassy Regional Oncology Dispensary, Department of Hematology | Central Ethics Commission of MoH of Ukraine Ethics Commission of Municipal institution “Cherkasy Regional Oncology Dispensary” |
|  | Dnepropetrovsk City Clinical Hospital #4, Regional Hematology Center | Central Ethics Commission of MoH of Ukraine Local Ethic Committee of Dnepropetrovsk City Clinical Hospital #4 |
|  | Institute of Urgent and Recovery Surgery named after V.K.Gusaka of AMS of Ukraine, Haematology Dept. | Bioethics Commission of Institute of Urgent and Recovery Surgery named after V.K.Gusaka of AMS of Uk |
| USA | Center For Cancer Care At Goshen Health System | Copernicus Group IRB |
|  | Hematology Oncology Associates Of Northern New Jersey | Sterling Institutional Review Board |
|  | Sinai Hospital | LifeBridge Health, Inc. |
|  | Oncology Hematology West, P.C. Methodist Cancer Center | Nebraska Methodist Hospital Institutional Review Board |
|  | Cancer Outreach Association | Sterling Institutional Review Board |
|  | Mid Dakota Clinic | Sterling Institutional Review Board |
|  | Columbia Comprehensive Cancer Care Clinics | Sterling Institutional Review Board |
|  | Diablo Valley Onc | Sterling Institutional Review Board |
